# VEGAWES: variational segmentation on whole exome sequencing for copy number detection

**DOI:** 10.1186/s12859-015-0748-0

**Published:** 2015-09-29

**Authors:** Samreen Anjum, Sandro Morganella, Fulvio D’Angelo, Antonio Iavarone, Michele Ceccarelli

**Affiliations:** 1 Computational Sciences and Engineering, Qatar Computing Research Institute, Doha, P. O. Box 5825 Qatar; 2 Department of Science and Technology, University of Sannio, Benevento, 82100 Italy; 3 European Molecular Biology Laboratory, European Bioinformatics Institute, (EMBL -EBI), Wellcome Trust Genome Campus, Cambridge, CB10 1SD UK; 4 BIOGEM, Ariano Irpino, 83031 Italy; 5 Institute for Cancer Genetics, Columbia University, New York, 10027 USA

**Keywords:** Copy number variation, Whole-exome sequencing, Segmentation, Variational based model

## Abstract

**Background:**

Copy number variations are important in the detection and progression of significant tumors and diseases. Recently, Whole Exome Sequencing is gaining popularity with copy number variations detection due to low cost and better efficiency. In this work, we developed VEGAWES for accurate and robust detection of copy number variations on WES data. VEGAWES is an extension to a variational based segmentation algorithm, VEGA: Variational estimator for genomic aberrations, which has previously outperformed several algorithms on segmenting array comparative genomic hybridization data.

**Results:**

We tested this algorithm on synthetic data and 100 Glioblastoma Multiforme primary tumor samples. The results on the real data were analyzed with segmentation obtained from Single-nucleotide polymorphism data as ground truth. We compared our results with two other segmentation algorithms and assessed the performance based on accuracy and time.

**Conclusions:**

In terms of both accuracy and time, VEGAWES provided better results on the synthetic data and tumor samples demonstrating its potential in robust detection of aberrant regions in the genome.

## Background

Structural variants are genomic rearrangements larger than 50 bp accounting for around 1 % of the variation among human genomes. Deletions, duplications, triplications, insertions, and translocations can all result in copy number variations (CNVs) [1]. CNVs correspond to the regions of the genome that have been deleted or duplicated on certain chromosomes. Various biological studies have shown a close association between chromosomal regions aberrant in copy number (CN) and diseases like tumor [1, 2], intellectual disability and autism [3, 4]. Due to its importance in the study of the molecular basis of tumor, several techniques have been recently implemented to detect CNVs. In the past, CNVs were detected on data collected using array comparative genomic gybridization (aCGH), Single-nucleotide polymorphism (SNP) and fluorescence in situ hybridization (FISH) techniques. However, the low resolution data in these preliminary methods prohibits the detection of short CNVs.

Recently, Next Generation Sequencing (NGS), a low cost and high throughput sequencing technique, has emerged as an effective alternative approach for DNA analysis including CNV detection [5]. NGS involves parallel sequencing of massive amounts of short DNA strands, also known as the reads, from randomly fragmented copies of a genome. Although, NGS gained popularity with the development of Whole Genome Sequencing (WGS) that enabled several large scale sequencing projects, the costs, computational complexity and effectiveness of WGS has consistently posed limitations. Researchers are typically interested in the coding regions of the genome, known as the exons, which comprise of 1 % of the whole human genome and is cost effective with respect to whole genome sequencing.

Detection of CNV from NGS data comprises of three steps: preprocessing of the data to remove biases in the data, segmentation of the data, and classification of amplification/deletion events. Segmentation is used to identify the breakpoints at which these aberrations are likely to occur along the genome. This step is important as it allows identification of critical regions in the genome which contributes towards the detection of various genetic diseases. Hence, there is a need of accurate, efficient, and robust segmentation algorithms.

Several tools have been implemented to detect CNVs detection on WGS data using state-of-the-art segmentation algorithms. ReadDepth [6] is one such commonly used software that primarily contributes towards the preprocessing of the WGS raw data, wherein a new model for data representation is introduced, and deploys the Circular Binary Segmentation (CBS) [7] algorithm for segmentation. SeqCNA [8] also focuses on preprocessing of the data and proposes a novel approach to remove GC bias, and then applies the Gain and Loss Analysis of DNA (GLAD) [9] segmentation algorithm. CNV-TV [10] uses a Total Variation (TV) approach to fit the data. It applies a piecewise constant function with the TV penalized least squares, and detects the plateau/basin in the signal that indicates a amplification/deletion event. The Schwarz information criterion (SIC) is used to find the optimal parameter to control the sensitivity and specificity.

Unlike the case of WGS, there are fewer publicly available tools that perform CNV detection on Whole Exome Sequencing (WES) data. ExomeCNV [11] is one of the earliest tools that processes WES data to detect CNV. It uses CBS algorithm and proposes an optimal computation of parameters to call out CNVs. EXCAVATOR [12] is a recently published tool which introduces three normalization steps based on median approach [13]. It then calls the heterogeneous shifting level model (HSLM), which is a modified version of the shiting level model (SLM) segmentation algorithm [14] to include the distance between exons, and applies the FastCall algorithm [15] for CNV classification. Control-FREEC [16] is another such tool that is flexible with calling CNVs on WGS and WES data with or without matched normal samples. VarScan2 [17] uses a heuristic approach to call CNVs and CBS algorithm for segmentation. In addition to these tools that require a paired sample for CNV detection, there are other set of tools that detect CNVs based on large samples cohorts. Copy Number Inference From Exome Reads (CONIFER) [18] and XHMM [19] are two commonly used tools for this purpose. While both are based on a similar idea, CONIFER exploits the singular value decomposition (SVD) model to detect rare CNVs, and XHMM uses the Hidden Markov model (HMM) model, based on the principal component analysis (PCA) approach, to find the principal sources of variation. However, both tools require sampling of large number of sequences for detection.

In this work, we focus on segmentation of WES data while considering the accuracy as well as the computational performance of the algorithm. There are very limited segmentation algorithms to specifically analyze WES data. In CBS, the data is represented as a sequence of random variables and the change points are the locations of copy number changes. These change points are iteratively localized until the copy numbers of adjacent regions are significantly different. CBS was initially developed to detect CNV on aCGH data and has been extensively used in the past for segmentation of WES data. However, it gives poor computational performance due to the recursive splits. HSLM, introduced in EXCAVATOR, specifically designed for WES data to include the distance between the exons, gives a higher precision rate due to the parameters used to determine the confidence levels in the selection of a CNV.

In recent years, variational-based approaches are being widely considered to solve segmentation. Most solutions are primarily based on the discontinuity-adaptive variational models proposed by Mumford and Shah [20] and Rudin et al. [21], which have been applied to segmentation of piecewise constant images. Variational models include minimization of an energy functional that attempts to find a solution such that the similarity between the computed segmentation and the observed data is increased while penalizing complex or irregular solutions. The measure of regularity is taken into account by a penalty term in the energy function that typically weighs the variability of the solution. In the Mumford and Shah model, the regularity is measured with the length of the boundary between regions. A regularization parameter controls the balance between the interpolation term and the regularity term.

With their initial accomplishment in addressing several image processing problems [22, 23], variational based models have been experimented and proved to be successful with segmentation on several kinds of DNA data [10, 24]. Besides CNV-TV, variational estimator for genomic aberrations (VEGA) [24] is one such segmentation algorithm that is based on the Mumford and Shah model and similar to the region growing segmentation algorithms introduced in Koepfler et al. [25]. It performs minimization of the energy function that allows the identification of breakpoints and penalizes the number of segments with a bottom-up approach. In this approach, it merges smaller regions into larger ones in a sequential manner. The regularization parameter is computed based on data-driven heuristics. This algorithm has been applied on CN segmentation of aCGH data and shown to perform robustly and accurately when compared with three other state-of-the-art segmentation algorithms (CBS, Ultrasome [26], and SMAP: Segmental Maximum A Posteriori approach [27]).

Here, we propose a variational segmentation algorithm, inspired by VEGA, to detect CNV on WES data: VEGAWES. We created a pipeline to apply variational based model on WES data and modified the segmentation algorithm to include WES properties. To preprocess the WES data, we remove bias using an approach similar to the one taken in EXCAVATOR tool. In addition, we enhance the original VEGA model by introducing the distance between exons as a feature in the computation of the parameters for the energy function minimization. In order to evaluate and validate our algorithm, we compare the performance of VEGAWES in terms of accuracy and robustness (time) with CBS and HSLM, and with the original model of VEGA. The experiments were conducted on synthetic data as well as real data, a 100 Glioblastoma Multiforme (GBM) paired primary tumor WES samples, downloaded from The Cancer Genome Atlas (TCGA) database.

## Methods

Read count (RC) is one of the commonly used approaches for CN detection on WES data [5, 28]. In this process, the WES data are first used to extract the RC for each exon. This is done by calculating the number of reads aligned to an exon. Once the average read coverage has been computed for each exon in the paired sample (tumor and normal), the data are preprocessed for bias removal. The basic idea behind RC approach is that the read count of the genomic region should be proportional to the CN profile of the region. However, the data contain several forms of bias that need to be removed. In this work, we apply a two-step bias removal approach - Mappability and GC Correction. The data are then represented as a log ratio (LR) signal of the tumor sample with respect to its matched normal. This list of LR values corresponding to each exon is the input to the segmentation algorithm, which uses these values to fragment the signal and eventually help in the identification of amplifications and deletions in the genome. In the following subsections, we will describe the details of the algorithm.

### Preprocessing

This is the first and vital step in WES data analysis. The raw data contain several bias sources which affects the copy number profiles, and hence requires preprocessing for mitigation or normalization. Although with the paired sample (tumor with a matched normal) approach, normalization against the control samples is known to reduce the bias, it has been shown that preprocessing is still necessary for better assessment [29, 30].

In our work, we use BAM files of paired tumor-normal samples as the input files. In general, the raw reads (DNA sequences) from WES raw data are first aligned to their location in the reference genome using alignment tools such as BWA [31] or BOWTIE [32], and finally compiled to a BAM format. In order to reduce the mappability bias in our analysis, we removed the reads that are mismatched or aligned to multiple regions. The average RC (ARC) for each exon is, then, computed as the number of reads aligned to an exon over the size of that exon (in bases):

$$ {ARC}_{i} = \frac{RC_{i}}{s_{i}} $$

where *R* *C*_*i*_ is the number of reads aligned to exon *i* and *s*_*i*_ is the size of that exon.

The next step is to remove the biological bias present in the data due to GC content. We observed that GC bias exists in our data and is in compliance with the analysis previously made in a similar work [12]. The average read coverage of the exons is the highest for values of GC content between 35 % and 65 %, and decreases for the other values. To address this correlation, we apply the median normalization approach described in [13] and implemented in the recent tool [12]. This approach successfully reduced the bias in our data as well.

Once the data have been preprocessed to remove biases and ARC values computed, we calculate the logarithm of the ratio of the tumor average read coverage and the matched normal average read coverage, which is known as the LR signal and represented as:

$${LR}_{i} = \log_{2} \frac{\overline{AR{C^{t}_{i}}}}{\overline{AR{C^{n}_{i}}}} $$

where $\overline {AR{C^{t}_{i}}}$ and $\overline {AR{C^{n}_{i}}}$ are the average read coverage of the tumor sample and the normal sample for exon *i* respectively after correction. This LR signal, in which each data point corresponds to an exon, is passed on for segmentation to detect CN breakpoints.

### Segmentation

We extend VEGA segmentation algorithm, originally developed to detect CNVs on aCGH data, for its application on WES data. This algorithm is based on the Mumford and Shah variational model [20]. According to this model, an observation signal *u*_0_, defined on the domain *Ω*, is partitioned into a set of disjoint connected components (*Ω*=*Ω*_1_∪*Ω*_2_∪…∪*Ω*_*n*_). The set of points on the boundary between the *Ω*_*i*_ is denoted as *Γ*. This partition is modeled such that the signal varies smoothly within a component and discontinuously between the disjoint components. This is also known as the problem of piecewise smooth approximation. The solution to this problem requires the derivation of an optimal approximation *u* of *u*_0_ while penalizing the complexity of the solution using a regularization parameter, *λ*. Here, we adopt the special case of Mumford and Shah energy functional for piecewise constant approximation, which is best suited for CNV segmentation. The optimal approximation is achieved by minimizing the following energy function which, in the original two-dimensional space, can be expressed as:

(1) $$ E(u, \Gamma) = \sum_{i}{\int_{\Omega_{i}} (u_{o} -u_{i})^{2}dx dy + \lambda |\Gamma|}  $$

where *u*_*i*_ is the mean value of *u*_*o*_ within each connected component *Ω*_*i*_.

VEGA adopts the one-dimensional version of the piecewise model; the data *D*∈IR^*n*^ are represented as a vector of size *n*, where *n* is the number of exons ordered by the genomic position. The segmentation divides the data vector into *M* connected regions, denoted as *R*, and is defined as a set of ordered positions *Γ*={*b*_1_,…,*b*_*M*+1_}. Each region *R*_*i*_ contains all exons between breakpoints {*b*_*i*_,*b*_*i*+1_}. Derived from the Mumford Shah model for one-dimensional data, the piecewise constant energy function to be minimized in order to find the optimal approximation is [24]:

(2) $$ E(u, \Gamma) = \sum_{i}{\int_{b_{i}}^{b_{i+1}} (u_{o} -u_{i})^{2}dx + \lambda M}  $$

where *λ* is the regularization parameter that determines the number of segmented regions.

In order to minimize this function, adjacent regions *R*_*i*_ and *R*_*i*+1_ are iteratively merged in a pyramidal manner to create larger segments and the reduction of the energy can be shown as:

(3) $$ E(u, \Gamma\backslash \{b_{i}\}) - E(u, \Gamma) = \frac{|R_{i}||R_{i+1}|}{|R_{i}| + |R_{i+1}|} ||u_{i}-u_{i+1}||^{2} - \lambda  $$

where, |*R*_*i*_| and *u*_*i*_ are the length (number of exons) and LR mean value of the *i*-th region, respectively, and ||.|| is the *L*_2_ norm and ∖ is the set difference. Following a greedy procedure, we start with a segmentation having *n* regions for each LR measure. Then, at each step we merge the pair of adjacent regions that upon merging yields the maximum decrease of the energy functional. Since *λ* decides the end of merging, choice of an appropriate value is crucial to ensure the quality of the final segmentation. In VEGA, the selection for *λ* at each merging step is done dynamically, depending on two factors - the length and LR mean values of the consecutive regions being considered for the merger. Hence, the cost of merging two regions *R*_*i*_ and *R*_*i*+1_, associated to a breakpoint *b*_*i*_, is computed as follows:

(4) $$ \hat{\lambda_{i}} = \frac{|R_{i}||R_{i+1}|}{|R_{i}|+|R_{i+1}|} ||u_{i}-u_{i+1}||^{2}  $$

The adjacent regions are merged and the *i*-th breakpoint removed if $\hat {\lambda _{i}} < \lambda $. If the condition is not satisfied any further, this implies the energy function has reached its minimum and no merging can proceed. Therefore, *λ* is updated to the smallest $\hat {\lambda _{i}} + \epsilon $ (close to zero) and the merging is continued. The sequence of *λ* values is monotonically increasing as it corresponds to the amount of decrease of the energy functional at each step in (eq. (3)). Here, we adopt a stopping criterion in such a way that the final segmentation is obtained when the increase in lambda stabilizes, and merging any further does not correspond to a significant decrease of the energy. The final stopping value of *λ* is based on the variability of the adjacent region (*λ* values) and the total variability of the data, *ν*. The resulting computation for the stopping criterion is *Δ* *λ*=*λ*_*l*+1_−*λ*_*l*_≤*β* *ν*, where *β* is a positive constant (ideal value = 0.5–0.7).

The WES data are sparse and consists of varied distances between exons. This characteristic is distinct from WGS data and affects the behavior of the WES data upon segmentation. Hence, we take this distance feature into consideration, similar to the approach taken in the development of EXCAVATOR, in the extended version of the original VEGA model.

The pyramid approach in VEGA merges two regions if the cost of merging them, $ \hat {\lambda _{i}} $, is low. As mentioned earlier, in the original model the two parameters considered to compute the cost are the relative lengths of the regions (|*R*_*i*_|−|*R*_*i*+1_|) and the difference of the LR values of both regions ||*u*_*i*_−*u*_*i*+1_||. In the new model, we add a third parameter that considers the local average distance between the exons within a region. We compute the local average for a region by taking the mean of the distances between consecutive exons in the region and express as:

(5) $$ d_{i} = \frac{\sum_{j=1}^{|R_{i}|-1}{|m_{j} - m_{j+1}|}}{|R_{i}|}  $$

where *m*_*j*_ is the midpoint of exon *j* in the region *R*_*i*_ on the genome. Then, the difference between the average distances of the two regions is calculated, to influence the merging condition (the cost function). If the difference between the two regions is small, the regions are expected to merge. That is, the cost of merging two regions is reduced if both are sparsely populated or are densely populated. On the other hand, if one region is sparsely populated as compared to the other, then the difference between the local averages is high which reduces the chances of merging. The idea is that two adjacent regions will merge with respect to their relative sparsity/density. Therefore, to include this factor, the original update formula for $\hat {\lambda _{i}}$ is adjusted with the third parameter that computes a weighted difference between the local average of the distances of exons for the two regions and can be expressed in the following way:

(6) $$ \hat{\lambda_{i}} = \frac{|R_{i}||R_{i+1}|}{|R_{i}|+|R_{i+1}|} ||u_{i}-u_{i+1}||^{2} + \alpha\log(|d_{i} - d_{i+1}|)   $$

where *d*_*i*_ and *d*_*i*+1_ are the averages of the distances between consecutive exons in *R*_*i*_ and *R*_*i*+1_ respectively, and *α* determines the weight of the parameter. In our analysis, we set the *α* variable to 0.001. This variable, when set to 0, also provides the flexibility of using the original VEGA model for segmentation on WES data.

The pseudocode of the resulting VEGAWES algorithm is reported below:



### Synthetic data

We generated synthetic chromosomes from the corrected ARC data of the eight samples described in [33]. The dataset consisted seven samples of Yoruba ancestry (NA19131, NA19138, NA19152, NA19153, NA19159, NA19206 and NA19223) and one sample of Caucasian ancestry (NA10847). We applied a similar procedure reported in [12] to generate these chromosomes using the seven samples as test and one sample as the control for each test sample.

Each synthetic chromosome consists of 1,000 exons and has *g* altered genes, where *N* is the length of an altered gene (in exons). The distance, in bp, between consecutive genes was defined as *D*. We performed tests on both amplification and deletion events separately and with several combinations of *g*, *N*, and *D*: g = (2, 5), *N* = (5, 20), and *D* = (10,000, 1,000,000). For each combination, we generated 100 synthetic chromosomes. In order to report the segmentation performance of each algorithm on the synthetic chromosomes, we used the receiver operating characteristic (ROC) curve as described in [34]. To compute ROC curve, true positive rate (TPR) was defined as the total number of exons in the altered regions whose segmented mean LR value is above the threshold divided by the total number of exons in the altered region and the false positive rate (FPR) was defined as the total number of exons in the unaltered regions whose segmented mean LR value is above the threshold divided by the total number of exons in the unaltered regions.

### Tumor dataset

In order to assess our algorithm on real data, we experimented with data provided by TCGA. All patient data were acquired from the published TCGA GBM Analysis project [35] in which it is stated that “Specimens were obtained from patients, with appropriate consent from institutional review boards” in accordance with the policies and guidelines outlined by the Ethics, Law and Policy Group from TCGA. All patient data is anonymous and was originally collected for routine therapeutic purposes.

The SNP data were used as ground truth for comparison and validation of the results generated by our algorithm. The reads in the BAM files were aligned to the Hg19 reference genome (ftp://ftp.ncbi.nlm.nih.gov/sra/reports/Assembly/GRCh37-HG19_Broad_variant/Homo_sapiens_assembly19.fasta) from the Broad Institute.

The sample sets comprised of varied coverage values. The total number of reads (RC) in the control samples ranges between 1.6–7.4 billion while in the case of the tumor samples the values range between of 3.4 – 7.9 billion. The total average reads per exon in the control samples lie between 7.2–33 million and the values for the tumor samples lie between 15–35 million. Furthermore, the average coverage per base in the normal samples range between 32.23–146.44, and 53.61–177.76 for tumor samples. The size of the exons in the reference genome varies between 24–91k bp and the average size is approximately 352 bp.

Prior to performing the evaluation, the data were preprocessed and the ARC values were generated using the DepthOfCoverage functionality of Genome Analysis Tool Kit (GATK-v3.2–2). The ARC values were then corrected for GC content, and the LR values were computed. The LR signal was then passed to each segmentation algorithm, and the results were obtained in the form of segments along with a corresponding mean LR value for each segment. In order to compare with the other algorithms, we set the parameters for each algorithm as per the default values provided by ExomeCNV for CBS and EXCAVATOR for HSLM.

We define amplification event as segments with LR value above 0.35 [36], deletion event as segments with values below -0.25 [37], and mark the rest as normal. In addition, since we focus on evaluating the results of the segmentation algorithms and not only the classified CNV labels, we also use the LR values of the exons computed by each algorithm with the corresponding SNP values for validation. The LR value of an exon is the mean LR value of the segment that contains the exon. An exon is marked as true positive if the copy number classification for the exon is the same as that in the SNP data or if the LR value from the segmentation algorithm is within the range of ±0.15 when compared with the LR value from the SNP data.

To analyze the accuracy of the segmentation on the tumor data, we use the precision, recall, and f-score metrics. We computed precision and recall for amplification and deletion events separately. For each sample, precision was defined as the ratio of the true positives detected by the algorithm that correctly correspond with the ground truth and the total number of regions detected by the algorithm. Recall was defined as the ratio of the true positives detected by the algorithm that correctly correspond with the ground truth and the total number of regions detected by SNP (ground truth). F-score, for each sample, is then the harmonic mean of the precision and recall scores.

## Results and discussion

To evaluate our segmentation algorithm, we analyzed the performance on both synthetic and real data. We compared our segmentation algorithm with two other commonly used and recently published algorithms: CBS (used in ExomeCNV, VarScan2, ReadDepth) and HSLM (EXCAVATOR). We also obtained results from the original VEGA model.

### Results on synthetic data

**Fig. 1:**
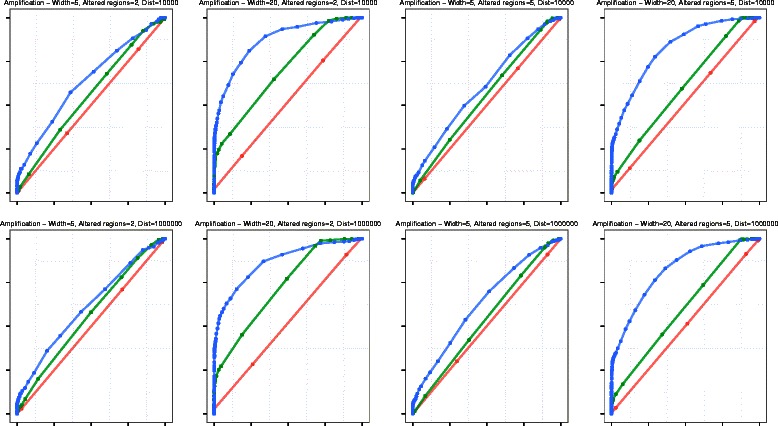
ROC Curves - Amplification: Comparison of segmentation performance of HSLM (green), CBS (red), and VEGAWES (blue) on synthetic data. The x-axis represents the False Positive Rate (1-Specificity) while the y-axis is the True Positive Rate (Sensitivity)

**Fig. 2:**
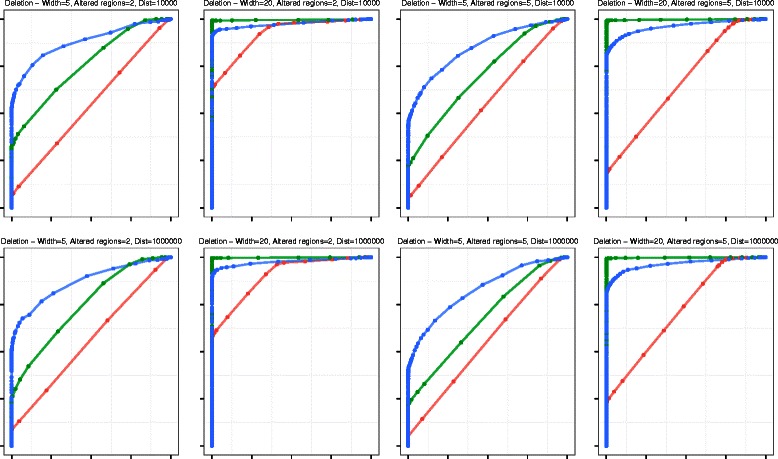
ROC Curves - Deletion: Comparison of segmentation performance of HSLM (green), CBS (red), and VEGAWES (blue) on synthetic data. The x-axis represents the False Positive Rate (1-Specificity) while the y-axis is the True Positive Rate (Sensitivity)

We performed an extensive simulation using 100 synthetic chromosomes, each comprising 1,000 exons. We computed ROC curves for VEGAWES, CBS and HSLM for each combination of *g*, *N*, and *D*, and demonstrate the results for amplification and deletions in Figs. 1 and 2 respectively. In general, considering both amplification and deletion events, we can observe that both VEGAWES and HSLM outperform CBS on synthetic data. As mentioned in [12], we also observe that overall, all algorithms perform better in deletion than amplification due the difference in signal-shifts. However, VEGAWES manifests better accuracy with amplification compared to other algorithms for all combinations of chromosomes. With regards to deletion, we notice that VEGAWES segments chromosomes with smaller altered regions more accurately than HSLM, while HSLM performs better on larger altered regions. Moreover, we observed that the distance between genes, *D*, does not seem to affect the accuracy of VEGAWES, and hence we report the results on *D* = 10,000 and *D* = 1,000,000 as extreme values.

### Results on Glioblastoma Multiforme dataset

**Fig. 3:**
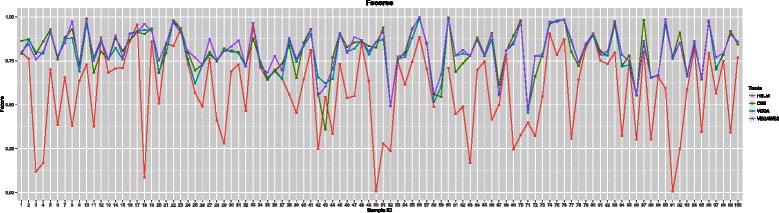
Fscore for each GBM sample: This figure illustrates the fscores computed on the results obtained by the four algorithms on 100 GBM samples

Glioblastoma Multiforme (GBM) is a particular malignant and aggressive type of brain tumor. We downloaded 100 GBM paired primary tumor WES samples in the BAM file format along with the corresponding control samples and the copy number profiles generated by the SNP array technology from TCGA. In Fig. 3, we illustrate the f-score for each sample and compare the four algorithms: HSLM, CBS, VEGA, and VEGAWES. The results obtained from CBS, VEGA, VEGAWES appear to be similar (*P*>0.1) whereas HSLM does not provide comparable results (*P*<10^−13^). We can further see in Table 1 the average f-scores for each tool, and observe that, although at a small margin, VEGAWES seems to perform the best in terms of accuracy. We have also listed the average precision and recall scores at the sample level, and notice that the scores obtained for gains and losses are the highest with VEGAWES. VEGA and CBS perform at a comparable rate with a mixed performance, while HSLM scores the lowest.

**Table 1:** Precision-recall scores for each segmentation algorithm averaged at the sample level

	HSLM	CBS	VEGA	VEGAWES
Precision (Gain)	0.6803	0.8269	0.8166	**0.8520**
Recall (Gain)	0.5652	0.8442	0.8289	**0.8501**
Precision (Loss)	0.6089	0.7387	0.7409	**0.7446**
Recall (Loss)	0.5192	0.8118	0.8174	**0.8157**
Fscore	0.5882	0.8029	0.7985	**0.8136**

The values highlighted in bold are the best scores achieved in the experiment

As a further analysis, we also compared the algorithms at the chromosome level. We computed the average precision-recall scores for the amplification and deletion events separately and have summarized in Table 2. In all four cases, we notice a similar performance at the chromosome level as we did at the sample level. VEGAWES provides better results followed by CBS, VEGA, and HSLM. Overall, from the two tables, we can state that VEGAWES performs marginally better than VEGA and CBS, while HSLM did not seem a good fit for this kind of dataset.

**Table 2:** Precision-recall scores for each segmentation algorithm averaged at the chromosome level

	HSLM	CBS	VEGA	VEGAWES
Precision (Gain)	0.5703	0.7460	0.7340	**0.7729**
Recall (Gain)	0.4891	0.7570	0.7404	**0.7709**
Precision (Loss)	0.5260	0.6705	0.6729	**0.6810**
Recall (Loss)	0.4644	0.7335	0.7410	**0.7422**

The values highlighted in bold are the best scores achieved in the experiment

**Fig. 4:**
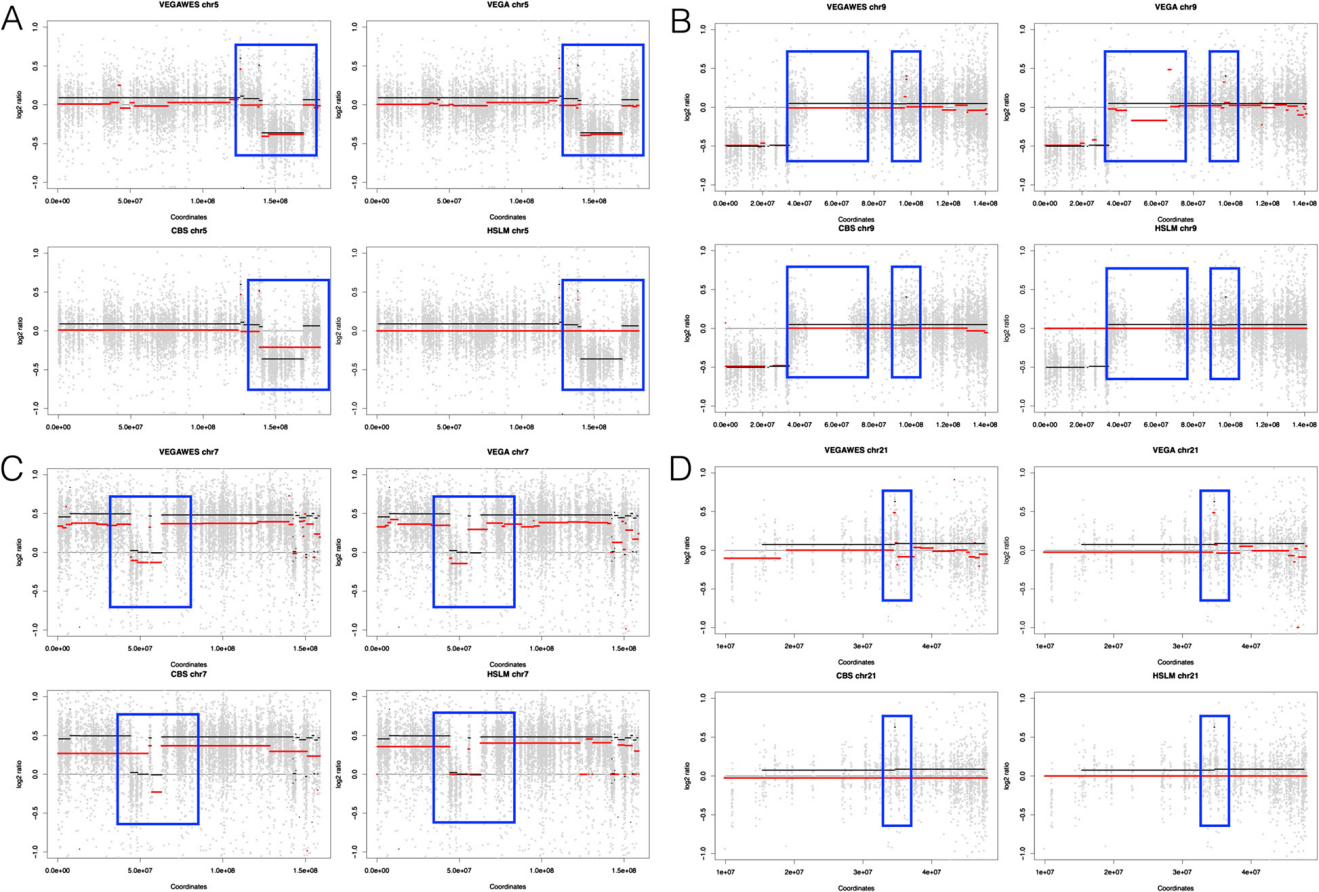
Segmentation results obtained from HSLM, CBS, VEGA, and VEGAWES respectively on TCGA samples. The black lines represent the ground truth (SNP data) while the red lines mark the segments obtained from the segmentation algorithms. **a** Chromosome 5 of sample set TCGA-41-2572, (**b**) Chromosome 9 of sample set TCGA-06-0124, (**c**) Chromosome 7 of sample set TCGA-12-0688, (**d**) Chromosome 21 of sample set TCGA-06-0129

To show the difference in accuracy between the four algorithms on real data, we have plotted a few examples of the segmentation results on the chromosomes of different sample sets. In Fig. 4, we plot the exons in the chromosome and the segmentation results obtained from the different tools as well as the SNP data. The black line represents the true segments of the SNP data while the red lines show the results from each algorithm. In Fig. 4 b, CBS, VEGA and VEGAWES provide better segmentations compared to HSLM. Specifically, VEGAWES performs better than VEGA showing that including the distance parameter improves the merging quality. Similarly, Fig. 4 c is yet another example where VEGAWES provides better segmentation compared to VEGA, CBS, and HSLM. This figure shows segmentation results on chromosome 7 which is one of the most frequently amplified chromosomes in GBM. Finally, in the last example (Fig. 4 d), although the regions are more granular when compared to the SNP segments, we observe that the VEGA and VEGAWES are both able to segment out the short amplified region unlike the other two counterparts.

**Fig. 5:**
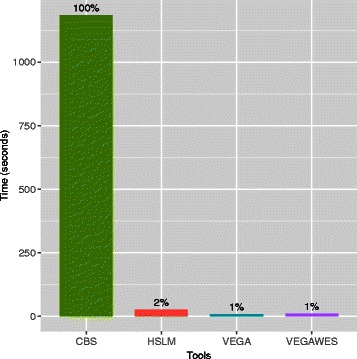
Computation Performance. Time taken to segment 22 chromosomes and averaged over 100 GBM samples for each algorithm

In addition to comparing the accuracy of the segmentation algorithms, we also compared the time performance on real data. All four segmentation algorithms were run on a x86_64 Linux platform with eight 2GHz quad-core CPUs. We recorded the time taken to segment all 22 autosomes for 100 samples and have summarized the results in the Fig. 5. In terms of time per sample, VEGA finishes first at 7.89 seconds followed very closely by VEGAWES at 8.286 seconds. On the contrary to the accuracy performance, HSLM performs better than CBS with regards to time. CBS takes about 19.75 minutes for each sample on average, which is considerably slow in comparison with the other three algorithms.

Considering both accuracy and time, VEGAWES and VEGA provide better results and takes the least time. While CBS provides similar segmentation results, it is about 143 times slower than VEGA and VEGAWES. On the other hand, HSLM takes less time to segment the chromosomes but fails at calling out several amplification and deletion events.

## Conclusion

We have developed a segmentation algorithm VEGAWES, based on Mumford and Shah variational model, to perform copy number segmentation on whole exome sequencing data. In our pipeline, we preprocess paired tumor-normal tumor BAM samples and prepare it for CN segmentation using VEGAWES. In addition to the variational approach derived from VEGA, we have enhanced the original model to include a specific property in WES data related to the average distance between exons of adjacent regions. VEGA follows a pyramidal approach where smaller segments are merged to create larger ones. In VEGAWES, we have taken the distance between exons into consideration while merging adjacent segments in addition to the existing parameters.

To validate our approach, we experimented our pipeline on both synthetic and real data of 100 GBM primary tumor samples and compared the segmentation results in terms of time performance and accuracy with two other algorithms commonly used in WES segmentation - CBS and HSLM. We also compare the results of VEGAWES with the original VEGA model. We observed that on synthetic data, VEGAWES outperforms both CBS and HSLM in all amplification events, whereas with deletion, it segments shorter regions more accurately than both other algorithms. With experiments on real data, we noticed that while VEGAWES provided similar or better segmentation results as compared to CBS, the time difference between the two approaches were significant with CBS performing very slow. Both VEGAWES and VEGA beat HSLM in terms of accuracy, although in terms of time, all three are comparable. We also reported that introducing the distance between the exons measure into the model reduced errors and detection of false positives in the segmentation results. However, we have included a weight parameter in the model that allows the usage of both the original VEGA model and the enhanced VEGAWES for segmentation purposes.

## Abbreviations

aCGH: Array comparative genomic hybridization
ARC: Average read count
CBS: Circular binary segmentation
CNV: Copy number variation
CN: Copy number
CONIFER: Copy number inference from exome reads
FISH: Fluorescence in situ hybridization
FPR: False positive rate
GATK: Genome analysis tool-kit
GBM: Glioblastoma multiforme
GLAD: Gain and loss analysis of DNA
HMM: Hidden Markov model
HSLM: Heterogeneous shifting level model
LR: Log-ratio
NGS: New generation sequencing
PCA: Principal component analysis
RC: Read count
ROC: Receiver operating curve
SIC: Schwarz information criterion
SLM: Shifting level model
SMAP: Segmental maximum a posteriori approach
SNP: Single-nucleotide polymorphism
SVD: Singular value decomposition
WES: Whole exome sequencing
WGS: Whole genome sequencing
TCGA: The cancer genome atlas
TPR: True positive rate
TV: Total variation
VEGA: Variational estimator for genomic aberrations
